# Transfer posterior error probability estimation for peptide identification

**DOI:** 10.1186/s12859-020-3485-y

**Published:** 2020-05-04

**Authors:** Xinpei Yi, Fuzhou Gong, Yan Fu

**Affiliations:** 1 National Center for Mathematics and Interdisciplinary Sciences, Key Laboratory of Random Complex Structures and Data Science, Academy of Mathematics and Systems Science, Chinese Academy of Sciences, Beijing, 100190 China; 2 School of Mathematical Sciences, University of Chinese Academy of Sciences, Beijing, 100049 China

**Keywords:** Proteomics, Mass spectrometry, Quality control, Posterior error probability, Local false discovery rate, Transfer learning

## Abstract

**Background:**

In shotgun proteomics, database searching of tandem mass spectra results in a great number of peptide-spectrum matches (PSMs), many of which are false positives. Quality control of PSMs is a multiple hypothesis testing problem, and the false discovery rate (FDR) or the posterior error probability (PEP) is the commonly used statistical confidence measure. PEP, also called local FDR, can evaluate the confidence of individual PSMs and thus is more desirable than FDR, which evaluates the global confidence of a collection of PSMs. Estimation of PEP can be achieved by decomposing the null and alternative distributions of PSM scores as long as the given data is sufficient. However, in many proteomic studies, only a group (subset) of PSMs, e.g. those with specific post-translational modifications, are of interest. The group can be very small, making the direct PEP estimation by the group data inaccurate, especially for the high-score area where the score threshold is taken. Using the whole set of PSMs to estimate the group PEP is inappropriate either, because the null and/or alternative distributions of the group can be very different from those of combined scores.

**Results:**

The transfer PEP algorithm is proposed to more accurately estimate the PEPs of peptide identifications in small groups. Transfer PEP derives the group null distribution through its empirical relationship with the combined null distribution, and estimates the group alternative distribution, as well as the null proportion, using an iterative semi-parametric method. Validated on both simulated data and real proteomic data, transfer PEP showed remarkably higher accuracy than the direct combined and separate PEP estimation methods.

**Conclusions:**

We presented a novel approach to group PEP estimation for small groups and implemented it for the peptide identification problem in proteomics. The methodology of the approach is in principle applicable to the small-group PEP estimation problems in other fields.

## Background

Identification of the proteins expressed in cells or tissues plays an essential role in proteomics. In shotgun proteomics, proteins are first digested into peptide mixture that is then analyzed via high-throughput tandem mass spectrometry (MS/MS), resulting in thousands to millions of MS/MS spectra in a typical experiment. Analysis of these spectra leads to a great number of candidate identifications of peptides. Protein sequences are inferred from reliably identified peptides, followed by qualitative or quantitative analysis. The peptide identification based on MS/MS has become one of the key problems in proteomics [1, 2].

To identify the peptides, the MS/MS spectra are commonly searched against a protein sequence database. For each spectrum, candidate peptides from the database are scored according to the quality of their matches to the spectrum. The top scored peptide-spectrum match (PSM) is taken as a candidate peptide identification. However, for many reasons, e.g. the incompleteness of the protein database or the imperfectness of the scoring function, the top-scored PSMs are not always correct identifications. Thus, filtering and quality control of PSMs after database search is necessary [3].

The scores of correct PSMs are usually higher in trend than those of incorrect PSMs, but they always have an overlap, resulting the difficulty in recognizing the correct PSMs. In early years, a simple way was to specify an empirical threshold and consider the PSMs with scores higher than the threshold as the correct ones. However, such threshold may not be appropriate, resulting in reduced accuracy or sensitivity of peptide identification. Thus, a quality control method that not only ensures the identification accuracy, but also does not sacrifice the identification sensitivity is needed. Quality control of PSMs can be dealt with as a multiple hypothesis testing problem [4, 5]. Each PSM corresponds to a hypothesis test. The null hypothesis (*H*_0_) is that the peptide is incorrectly identified, and the corresponding alternative hypothesis (*H*_1_) is that the peptide is correctly identified. The most commonly used statistical confidence measure in multiple hypothesis testing is the false discovery rate (FDR) proposed by Benjamini and Hochberg [6]. FDR is defined as the expected proportion of incorrect ones among all rejections of null hypotheses.

At present, the common way to estimate the FDR of PSMs in proteomics is the target-decoy database search approach [7]. The principle of the target-decoy approach is simple: the experimental MS/MS spectra are searched against a database which not only consists of the target protein sequences but also the same size of decoy protein sequences (typically the reverse sequences of the target proteins). Because an incorrect identification has an equal chance of being a match to the target sequences or to the decoy sequences, the number of decoy PSMs can be used as an estimate of the number of false target PSMs and the FDR of target PSMs can be estimated by the ratio of decoy PSMs to the target PSMs above the score threshold.

FDR measures the global confidence of a collection of PSMs with different scores, whereas one may be interested in the confidence of PSM(s) with a specific score. The posterior error probability (PEP, also known as local false discovery rate) is defined as the probability of a hypothesis being null given the test statistic, and consequently it can measure the confidence of individual tests [8]. In our case, the PEP of a PSM is the probability that this PSM is incorrect given its score. Let *f*(*x*)=*π*_0_*f*_0_(*x*)+*π*_1_*f*_1_(*x*) denote the probability density function (pdf) of the scores of a collection of PSMs, with *f*_0_(*x*) being the pdf of the scores of incorrect PSMs, *f*_1_(*x*) the pdf of scores of correct PSMs, *π*_0_ the proportion of incorrect PSMs, and *π*_1_=1−*π*_0_. Bayes’ rule gives,

1 $$\begin{array}{@{}rcl@{}} \text{PEP}(x) &=& \text{Prob}(H_{0}|x)=\frac{\pi_{0}f_{0}(x)}{f(x)}  \end{array} $$

FDR can be derived from PEP using a simple relationship between them, i.e., FDR(*x*)=E_*f*_{PEP(*s*)|*s*≥*x*}. Therefore, whenever possible, estimation of PEP is always more desirable than FDR.

PEP estimation relies on decomposing the mixture distribution of *f*(*x*). There are three approaches to achieve this aim in proteomics: parametric, semi-parametric, and non-parametric approaches. The early PeptideProphet [9] algorithm was a parametric approach, in which *f*_0_(*x*) and *f*_1_(*x*) are assumed to be specific types of distributions and their parameters are estimated from the observed scores using the EM (Expecting Maximization) algorithm. However, the parametric approach could be problematic if the assumption on the distribution types is inappropriate [2]. In addition, PeptideProphet did not make use of any decoy information to estimate *f*_0_(*x*). In the improved version of PeptideProphet [10, 11], *f*_0_(*x*) is first derived directly from the scores of decoy PSMs using kernel density estimation, and then *f*_1_(*x*) and *π*_0_ are estimated using a semi-parametric method [12]. This semi-parametric and semi-supervised approach is more flexible and stable. Different from PeptideProphet, which estimates *f*_0_(*x*) and *f*_1_(*x*) explicitly, the method proposed by Käll et al. [13] estimates $\frac {f_{0}(x)}{f(x)}$ directly with a non-parametric approach and estimates *π*_0_ by bootstrap.

In proteomics, it is often the case that only a group (subset) of peptide identifications, e.g. those with specific post-translational modifications (PTMs) or from specific proteins, are focused on [14–17]. Thus, group FDR estimation is necessary. The most straightforward way to estimate the FDR of the group is to simply use the combined FDR estimated on all PSMs as the FDR for the PSM group of interest. However, due to the difference between the score distributions of the group and the whole set of PSMs, the combined FDR may be greatly different from the real group FDR at the same score threshold, leading to unreliable or failed quality control of peptide identifications in the group [14, 18, 19]. Estimating the group FDR separately on the group PSMs is certainly a better choice, which we name the separate FDR estimation method. However, for small groups, the number of PSMs in the group may not be sufficient for reliable estimation of the separate FDR, leading to overly conservative or liberal FDR estimation, especially for higher-score interval where observed decoy PSMs are even fewer [20–22].

Fu et al. [21] proposed the transfer FDR method for quality control of small groups of peptide identifications. Transfer FDR derives the group FDR from the combined FDR based on the relationship between them. A key component of transfer FDR is to fit the proportion of decoy PSMs belonging to the group as a function of PSM score, and extrapolate it to the high-score interval for group FDR estimation. Zhang et al. [23] and Li et al. [24] developed methods of similar rationales but less rigors in estimating the proportion of group decoy PSMs.

It is also desirable to evaluate the PEPs of individual PSMs in the group of interest. Similar to the case of FDR, two direct methods can be used to estimate the group PEP, i.e., the *combined PEP* (estimate the group PEP using the whole set of PSMs) and the *separate PEP* (estimate the group PEP solely using the PSMs in the group). However, these two methods have the same problems faced by combined FDR and separate FDR as mentioned above. Especially, when the group is very small, separate PEP estimation is even infeasible.

As far as we know, there are currently no group PEP estimation methods for small groups in proteomics and there are few in statistics. Efron [18] discussed the necessity of group PEP estimation and proposed a general approach, named class-wise fdr, based on the relationship between the group PEP and the combined PEP in the Bayesian framework. In order to calculate the relationship, class-wise fdr supposes the cases in the group under *H*_0_ come from a normal distribution, which, however, may not hold in some applications, e.g. peptide identification.

Here, we present a group PEP estimation method, named *transfer PEP*, for quality control of small groups of peptide identifications. Inspired by the transfer learning technology [25], which transfers the knowledge from one domain to another domain for better learning with insufficient training data, transfer PEP builds on the empirical relationship between the group distribution and the combined distribution of PSM scores. When the group null distribution is different from the combined counterpart, transfer PEP derives it from the fitted proportion of group decoy PSMs among all decoy PSMs. When the group alternative distribution is different from the combined counterpart, transfer PEP estimates it, as well as *π*_0_, using a semi-parametric method. The accuracy and power of transfer PEP were validated on simulated data and real MS/MS data of peptides.

## Algorithm

The aim is to estimate PEP_*G*_(*x*), the PEP of PSMs in a group *G* at arbitrary score *x*:

2 $$\begin{array}{@{}rcl@{}} \text{PEP}_{G}(x) &=& \text{Prob}(H_{0}|x,G)=\frac{\pi_{G0}f_{G0}(x)}{\pi_{G0}f_{G0}(x)+\pi_{G1}f_{G1}(x)}  \end{array} $$

where *f*_*G*0_(*x*) and *f*_*G*1_(*x*) are the pdfs of null and alternative distributions of group *G*, i.e. the pdfs of the scores of incorrect and correct PSMs in the group, respectively, *π*_*G*0_ is the proportion of incorrect PSMs in the group, and *π*_*G*1_=1−*π*_*G*0_.

We deal with the situation in which the group *G* is so small that *f*_*G*0_(*x*), *f*_*G*1_(*x*) and *π*_*G*0_ cannot be estimated directly. We assume that the whole set of PSMs is always large enough such that *f*_0_(*x*), *f*_1_(*x*) and *π*_0_ can be accurately estimated out, e.g., using the same algorithm as in PeptideProhpet. The rationale of our algorithm, transfer PEP, is to make use of the relationship between the group and combined score distributions to help estimate PEP_*G*_(*x*).

### Estimation of *π*_*G*0_*f*_*G*0_(*x*)

When *f*_*G*0_=*f*_0_, *f*_0_ is directly used as *f*_*G*0_. When *f*_*G*0_≠*f*_0_, we establish a relationship between them as follows. Define *γ*_*G*_(*x*)=Prob(*G*|*H*_0_,*s*≥*x*), where *s* is the PSM score. As we previously showed, *γ*_*G*_(*x*) can be readily fitted as a linear function of *x* using decoy PSMs, the given incorrect PSMs [21]. Let *F*_0_(*x*) and *F*_*G*0_(*x*) denote the cumulative distribution functions (cdfs) of *f*_0_(*x*) and *f*_*G*0_(*x*), respectively. Bayes’ rule gives,

3 $$\begin{array}{@{}rcl@{}} \gamma_{G}(x) &=& \text{Prob}(G|H_{0},s\geq{x})\\ &=& \frac{\text{Prob}(G,H_{0})\text{Prob}(s\geq{x}|G,H_{0})}{\text{Prob}(H_{0})\text{Prob}(s\geq{x}|H_{0})}\\ &=& \frac{\text{Prob}(G,H_{0})(1-{F}_{G0}(x))}{\text{Prob}(H_{0})(1-{F}_{0}(x))}\\ &=& \frac{\pi_{G}\pi_{G0}(1-{F}_{G0}(x))}{\pi_{0}(1-{F}_{0}(x))}  \end{array} $$

Thus,

4 $$\begin{array}{@{}rcl@{}} \pi_{G0}(1-F_{G0}(x)) &=& \frac{\pi_{0}(1-F_{0}(x))\gamma_{G}(x)}{\pi_{G}}  \end{array} $$

Taking the derivatives of both sides of Eq. (4), we have

5 $$\begin{array}{@{}rcl@{}} \pi_{G0}f_{G0}(x) &=& {\frac{-\pi_{0}{(\gamma_{G}{(x)})}'(1-F_{0}(x))+\pi_{0}{\gamma_{G}{(x)}}f_{0}{(x)}}{\pi_{G}}}  \end{array} $$

where *π*_*G*_ is the ratio of group PSMs to all PSMs, which can be directly calculated.

### Estimation of *f*_*G*1_(*x*) and *π*_*G*0_

When *f*_*G*1_=*f*_1_, *f*_1_ is directly used as *f*_*G*1_. When *f*_*G*1_≠*f*_1_, there is no established relationship available between them, and we estimate *f*_*G*1_(*x*) and *π*_*G*0_ using a semi-parametric approach [10, 12]. In this approach, *f*_*G*1_(*x*) and *π*_*G*0_ are updated iteratively with an EM-like procedure. When *f*_*G*0_=*f*_0_ and *f*_*G*1_=*f*_1_, *π*_*G*0_ is the only parameter that needs to be estimated. In this case, we estimate it using the same iterative procedure, which reduces to a standard EM algorithm in the simplest form.

Algorithm 1 outlines the main steps of our transfer PEP algorithm. In the algorithm, the probability for each of the *n* group PSMs being correct is stored in a *n*-dimensional vector, *θ*_*G*_. In each iteration, *π*_*G*1_ is estimated by the average of *θ*_*G*_. *f*_*G*1_(*x*) is estimated by Gaussian kernels, *K*(·), with *θ*_*G*_ used as weights. Then, *θ*_*G*_ is updated using the current *π*_*G*1_, *f*_*G*1_(*x*), and *π*_*G*0_*f*_*G*0_(*x*). The above procedure is repeated until *θ*_*G*_ becomes stable.

### Equality judgement

In order to use the algorithm, we need to judge whether *f*_*G*0_=*f*_0_ and *f*_*G*1_=*f*_1_ in practice. Define *λ*_*G*_(*x*)=Prob(*G*|*H*_1_,*s*≥*x*). Then, we have the following two conclusions: (1) *f*_*G*0_=*f*_0_ if and only if *γ*_*G*_(*x*) is a constant, and (2) *f*_*G*1_=*f*_1_ if and only if *λ*_*G*_(*x*) is a constant. Take *γ*_*G*_(*x*) as an example. If *γ*_*G*_(*x*) is a constant *γ*, then by using Eq. (5), we have $f_{G0}(x)=\frac {{\pi _{0}}\gamma {f_{0}(x)}}{\pi _{G}\pi _{G0}}=Cf_{0}(x)$, in which *C* is a constant. Because *F*_*G*0_(*∞*)=*CF*_0_(*∞*)=1, *C*=1. Thus, *f*_*G*0_=*f*_0_. On the other hand, when *f*_*G*0_=*f*_0_, $\gamma _{G}(x)=\frac {\pi _{G}\pi _{G0}}{\pi _{0}}$, which is a constant.

Whether *γ*_*G*_(*x*) is a constant can be judged by examining whether the fitted *γ*_*G*_(*x*) is a horizontal line. Similar to *γ*_*G*_(*x*), *λ*_*G*_(*x*) can be estimated by the proportion of correct matches belonging to the group:

6 $$\begin{array}{@{}rcl@{}} \hat{\lambda}_{G}(x) &=& \frac{N_{Gt}(x)(1-\text{FDR}_{G}(x))}{N_{t}(x)(1-\text{FDR}(x))}\\ &=& \frac{N_{Gt}(x)-N_{Gd}(x)}{N_{t}(x)-N_{d}(x)}  \end{array} $$

where FDR_*G*_(*x*) is the group FDR at score threshold *x*, *N*_*Gt*_(*x*) is the number of target PSMs in the group with scores >*x*, *N*_*Gd*_(*x*) is the number of decoy PSMs in the group with scores >*x*, *N*_*t*_(*x*) is the number of target PSMs with scores >*x*, and *N*_*d*_(*x*) is the number of decoy PSMs with scores >*x*. At varying *x*, we calculate the estimated value of *λ*_*G*_(*x*), and examine whether or not these values approximate some constant.


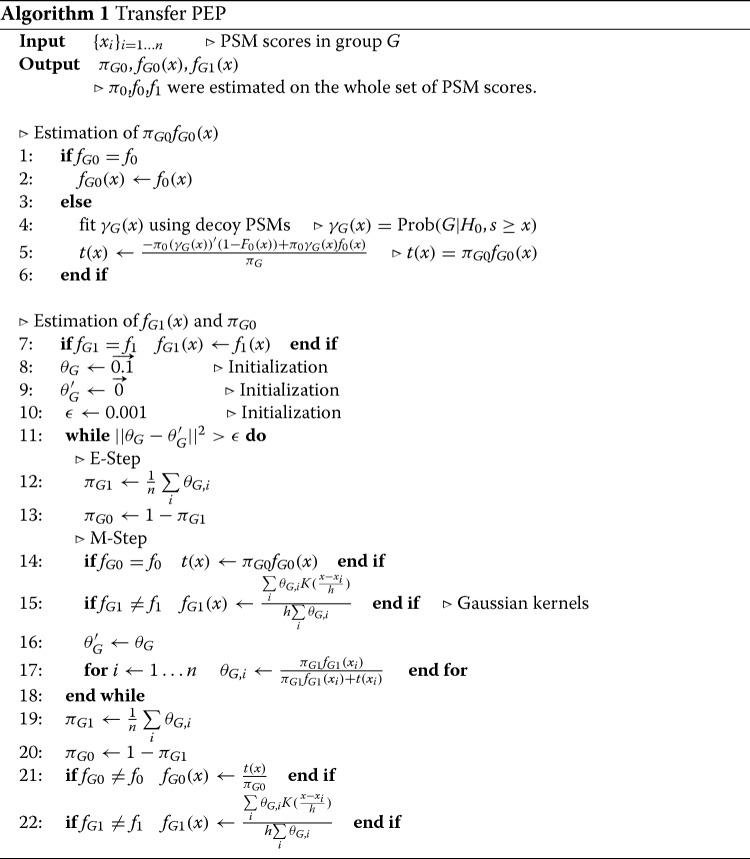


## Results

In order to validate the performance of the transfer PEP algorithm, we must be able to know the theoretical distribution of data so as to compare the estimated PEP to the theoretical PEP. However, the theoretical distribution is in general absent in the problem of peptide identification. Therefore, we prepared three different types of data to evaluate the accuracy and power of transfer PEP: (i) theoretical simulated data, (ii) simulated MS/MS data of peptides, and (iii) real MS/MS data of peptides.

Three methods for estimating the group PEP of peptide identifications were compared: combined PEP, separate PEP and transfer PEP. Combined PEP and separate PEP were estimated on the whole set of PSMs and on the PSMs in the group only, respectively, using the semi-parametric method as used in the PeptideProphet algorithm [10]. Transfer PEP was estimated using Algorithm 1 as described in the previous section.

Two criteria were used for evaluation: the consistency between the estimated PEP and the theoretical PEP, and the consistency between the estimated FDR and the real FDR. The estimated FDR was obtained by integration of the estimated PEP, and was used for evaluation on MS/MS data because the theoretical PEP was not available for them. The integrals of combined PEP, separate PEP and transfer PEP are denoted as iCombined FDR, iSeparate FDR and iTransfer FDR, respectively. Note that iTransfer FDR is not the transfer FDR which we proposed previously [21].

### Theoretical simulated data

To evaluate the consistency between the estimated PEP and the theoretical PEP, we simulated sets of scores for the case *f*_*G*0_≠*f*_0_ and *f*_*G*1_≠*f*_1_ under the condition that *γ*_*G*_(*x*)=*ax*+*b*, in which *a*≠0 and *b*≠0. All the scores were divided into two complementary groups: *G* and *Q*. Assume all the scores are greater than or equal to 0. From Eq. (4) we have $\pi _{G0}=\frac {b\pi _{0}}{\pi _{G}}$. Bringing it into Eq. (5) yields

7 $$\begin{array}{@{}rcl@{}} f_{G0}(x) &=& \frac{-a(1-{F}_{0}(x))+(ax+b)f_{0}(x)}{b}  \end{array} $$

According to the definition of *γ*_*G*_(*x*), we have Prob(*G*|*H*_0_)=*γ*_*G*_(0)=*b*, and Prob(*Q*|*H*_0_)=1−*b*. Because *f*_0_(*x*)=Prob(*G*|*H*_0_)*f*_*G*0_(*x*)+Prob(*Q*|*H*_0_)*f*_*Q*0_(*x*), we have

8 $$\begin{array}{@{}rcl@{}} f_{Q0}(x) &=& \frac{f_{0}(x)-bf_{G0}(x)}{1-b}  \end{array} $$

Thus if *γ*_*G*_(*x*)=*ax*+*b* and *f*_0_(*x*) are given, both *f*_*G*0_(*x*) and *f*_*Q*0_(*x*) are given as well.

In the simulation, we set *γ*_*G*_(*x*)=−0.01*x*+0.4 and *f*_0_(*x*)=*Gamma*(*x*,0.96,1.5), and derived *f*_*G*0_(*x*) and *f*_*Q*0_(*x*) using Eq. (7) and Eq. (8), respectively. The total number of scores were *N*=15000. The proportion of incorrect scores (from null distribution *f*_0_) was *π*_0_=0.65. Among the *N*_0_ incorrect scores, *N*_*G*0_ scores were generated from *f*_*G*0_(*x*) with probability Prob(*G*|*H*_0_)=*b*=0.4, and *N*_*Q*0_=*N*_0_−*N*_*G*0_ scores were generated from *f*_*Q*0_(*x*) with probability Prob(*Q*|*H*_0_)=1−*b*=0.6. Among the *N*_1_=*N*−*N*_0_ correct scores (from alternative distribution *f*_1_), *n* (=1, 10, 20, 50, 100) scores were generated from *f*_*G*1_(*x*)=*N*(9,6) and *N*_1_−*n* scores were generated from *f*_*Q*1_(*x*)=*N*(10,6). The choice of gamma and normal distributions to generate the incorrect and correct scores is because they resemble the real distributions [10, 26]. To mimic the target-decoy strategy, *N*_0_ decoy scores were generated. Among them, *N*_*G*0_ scores were from *f*_*G*0_(*x*) and *N*_*Q*0_ scores were from *f*_*Q*0_(*x*). This simulation was repeated *S*=1000 times.

**Fig. 1:**
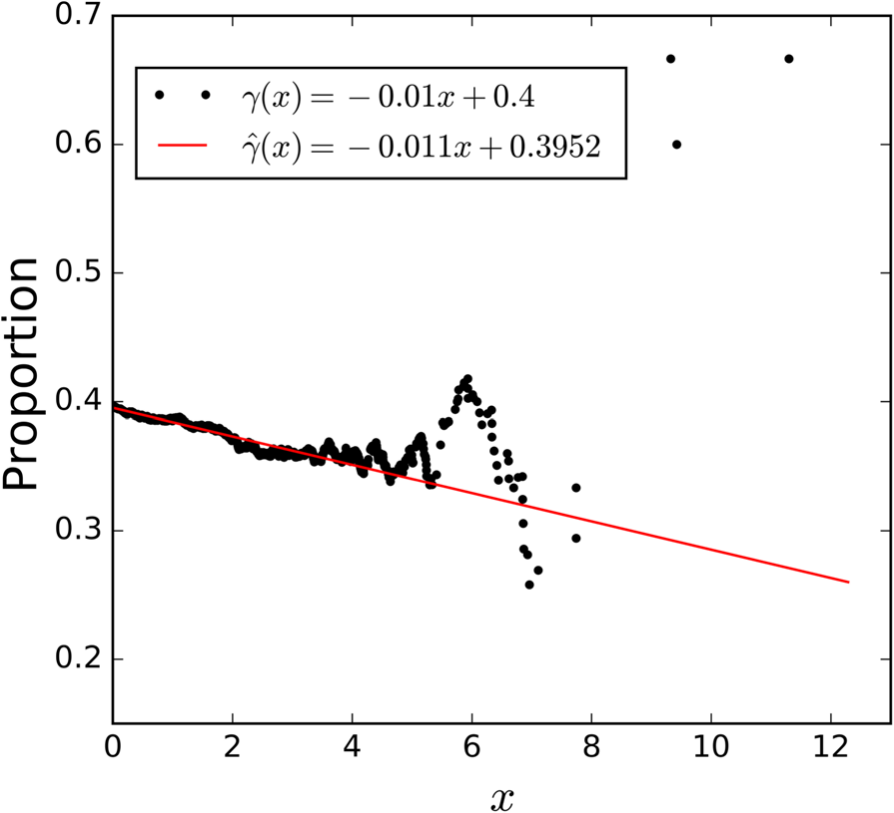
The linear fitting result of *γ*_*G*_(*x*) on the simulated data. The x-axis represents the score threshold and y-axis represents the proportion of decoy scores belonging to group *G* above the threshold

*γ*_*G*_(*x*) was fitted as a linear function using the observed proportions of decoy scores belonging to group G above threshold *x*, as shown in Fig. 1. Notice that big deviation was observed at critical regions, i.e. large scores, which correspond to small FDRs and we care the most. This deviation was caused by the random fluctuation of the proportion calculated from very limited number of scores. The similar phenomenon was observed on MS/MS data (Figs. 3, 5 and 8). The proportions for large scores should be extrapolated from the fitted function. This is the very rational of transfer PEP.

**Fig. 2:**
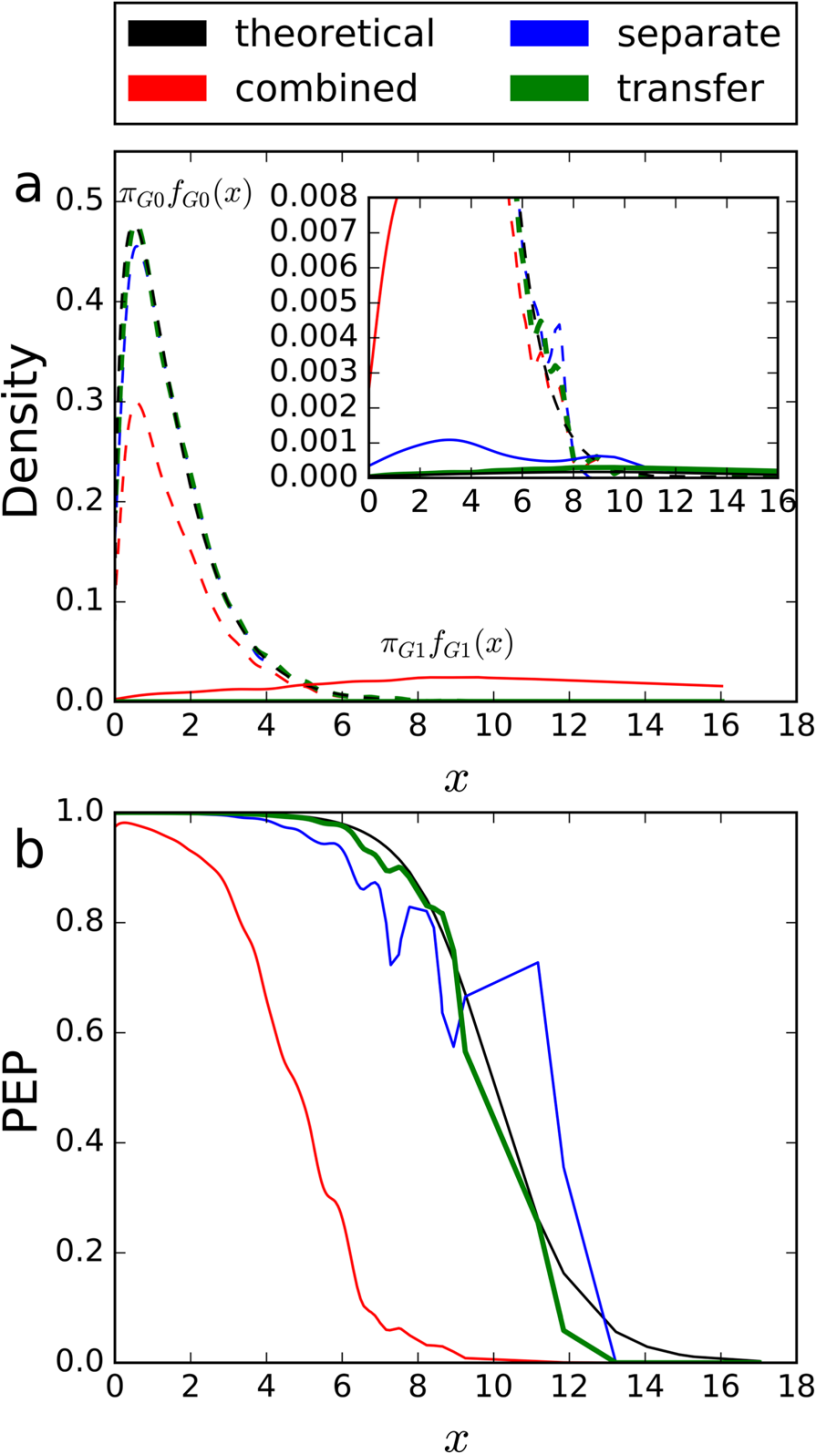
Example of results of the three PEP estimation methods in one simulation. **a** Estimated and theoretical distributions. The dotted line represents *π*_*G*0_*f*_*G*0_(*x*) and the solid line represents *π*_*G*1_*f*_*G*1_(*x*). **b** Estimated and theoretical PEPs

Figure 2 shows the results of the three PEP estimation methods in one simulation in which the number of scores from *f*_*G*1_(*x*) is *n*=10. As shown in Fig. 2a, both *π*_*G*0_*f*_*G*0_(*x*) and *π*_*G*1_*f*_*G*1_(*x*) estimated by combined PEP seriously deviated from the theoretical distributions. The result of separate PEP was much better, but still had significant deviations at some regions due to the insufficient sample size. Benefiting from the estimation of *γ*_*G*_(*x*), transfer PEP gave remarkably accurate estimates of both *π*_*G*0_*f*_*G*0_(*x*) and *π*_*G*1_*f*_*G*1_(*x*). The group PEP curve estimated by the transfer PEP was also the most accurate among the three methods, as shown in Fig. 2b.

**Fig. 3:**
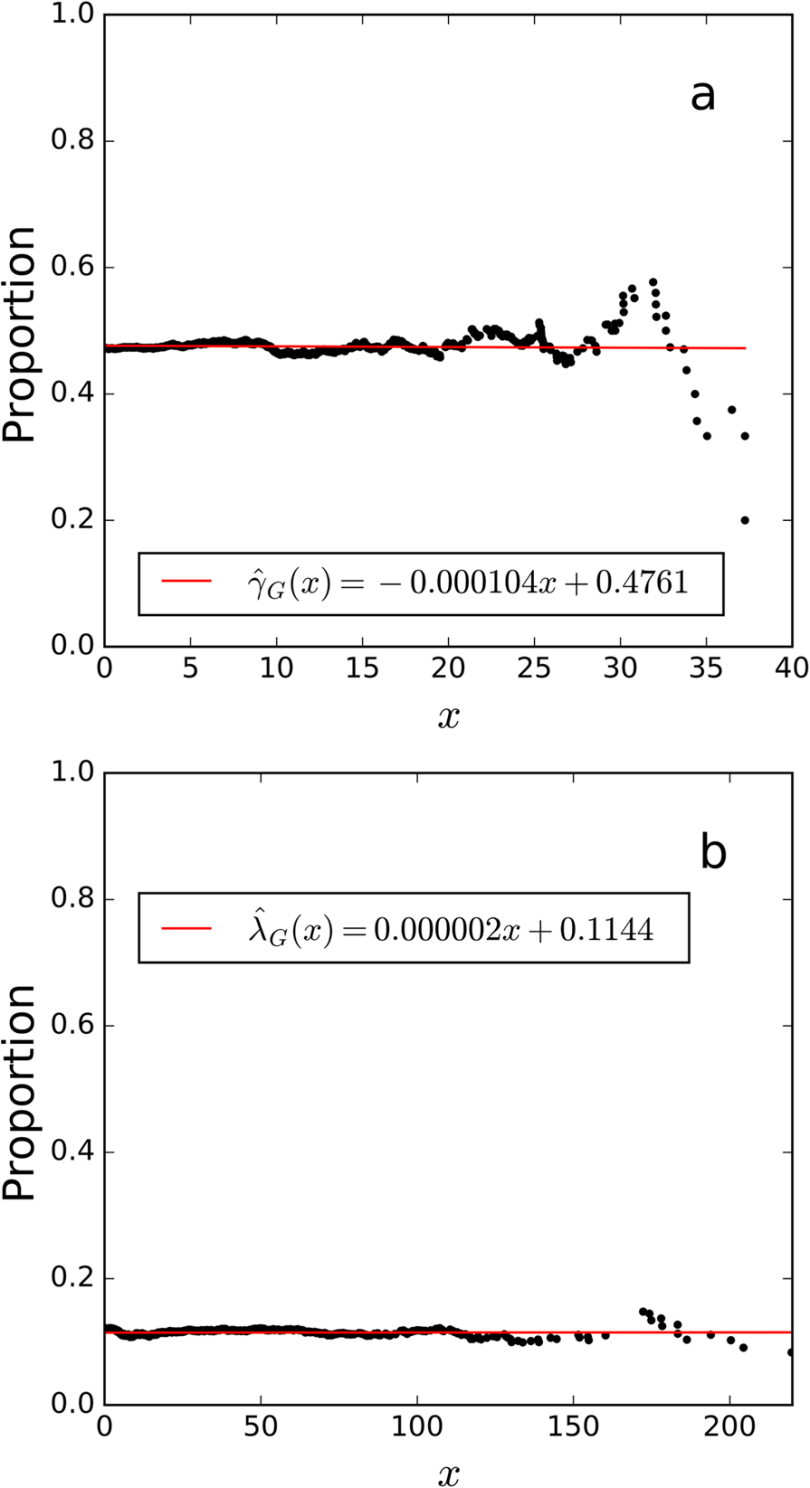
Examples of linear fitting results of **a** *γ*_*G*_(*x*) and **b** *λ*_*G*_(*x*) in the variant-peptide identification simulation study. The x-axis represents the score threshold, and the y-axis represents **a** the proportion of decoy variant PSMs among all decoy PSMs, or **b** the estimated proportion of correct variant PSMs among all variant PSMs above the score threshold *x*

To evaluate the average performance of each estimation method in the *S* simulations, we calculated the mean and standard deviation (SD) of mean squared error (MSE) between the estimates, $\hat {\text {PEP}}_{G}$, and the theoretical values, PEP_*G*_, for top scores (*Ratio*=1*%*,5*%*,10*%*,20*%*,100*%*). The MSE in the *j*^*th*^ simulation for the given values of *Ratio* and *n* (the number of correct scores generated from *f*_*G*1_(*x*)) is calculated as:

$$\text{MSE}_{j}(n,{Ratio})=\frac{1}{N_{j}}\sum\limits_{i=1}^{N_{j}}\left(\hat{\text{PEP}}_{G,i,j}-\text{PEP}_{G,i,j}\right)^{2} $$

where *N*_*j*_ denotes the number of top *Ratio* scores in the *j*^*th*^ simulation, and $\hat {\text {PEP}}_{G,i,j}$ and PEP_*G*,*i*,*j*_ denote the estimated and theoretical PEPs of the *i*^*th*^ score in the *j*^*th*^ simulation, respectively. Then, we compute the mean and SD of MSEs over the *S* simulations as:

$$\text{Mean}(n,{Ratio})=\frac{1}{S}\sum\limits_{j=1}^{S}\text{MSE}_{j}(n,{Ratio}) $$

$$\text{SD}(n,{Ratio})=\sqrt{\frac{1}{S}\sum\limits_{j=1}^{S}(\text{MSE}_{j}(n,{Ratio})-\text{Mean(n,{Ratio})})^{2}} $$

The quality of the estimates provided by the three estimation methods in the configuration (*n*,*Ratio*) is measured by both Mean(*n*,*Ratio*) and SD(*n*,*Ratio*).

Table 1 shows the results. When the number of scores from *f*_*G*1_(*x*) was small (*n*=1,10,20,50), both the mean and SD of MSE were very large for the combined PEP, especially for the high-score regions. The separate PEP was much better, but still deviated from the theoretical PEP_*G*_ when the number of scores from *f*_*G*1_(*x*) was too small (*n*=1,10,20), especially for the high-score regions. For all the configurations of *Ratio* and *n*, the transfer PEP estimated the PEP_*G*_ accurately. With increasing *n* and *Ratio*, the performances of both the combined PEP and the separate PEP gradually approached the performance of transfer PEP.

**Table 1:** The PEP estimation errors of three methods on the simulated data

	*Method*	*Ratio=1%*		*Ratio=5%*		*Ratio=10%*		*Ratio=20%*		*Ratio=100%*	
		*Mean*	*SD*	*Mean*	*SD*	*Mean*	*SD*	*Mean*	*SD*	*Mean*	*SD*
*n=1*	Combined	71.88	4.53	33.75	3.08	19.75	1.89	10.71	1.02	2.27	0.20
	Separate	7.02	5.67	1.81	1.46	0.98	0.80	0.52	0.43	0.11	0.09
	Transfer	**4.33**	**4.14**	**1.21**	**1.14**	**0.68**	**0.64**	**0.37**	**0.35**	**0.08**	**0.08**
*n=10*	Combined	56.71	3.54	31.91	2.75	18.93	1.76	10.31	0.96	2.19	0.19
	Separate	4.86	4.20	1.38	1.20	0.76	0.66	0.41	0.36	0.09	0.08
	Transfer	**2.78**	**2.70**	**0.89**	**0.90**	**0.51**	**0.52**	**0.28**	**0.29**	**0.06**	**0.06**
*n=20*	Combined	41.11	3.75	29.73	2.59	18.00	1.74	9.87	0.98	2.11	0.19
	Separate	3.78	3.41	1.24	1.13	0.70	0.65	0.38	0.36	0.08	0.08
	Transfer	**2.00**	**1.88**	**0.76**	**0.81**	**0.46**	**0.49**	**0.26**	**0.28**	**0.06**	**0.06**
*n=50*	Combined	8.41	3.62	25.67	2.14	16.36	1.53	9.10	0.86	1.95	0.17
	Separate	2.04	1.51	1.02	0.90	0.59	0.54	0.33	0.31	0.07	0.07
	Transfer	**1.01**	**0.81**	**0.66**	**0.70**	**0.41**	**0.44**	**0.23**	**0.26**	**0.05**	**0.06**
*n=100*	Combined	0.03	0.05	19.10	1.68	13.77	1.36	7.87	0.78	1.70	0.15
	Separate	0.13	0.23	0.80	0.66	0.47	0.41	0.26	0.23	0.06	0.05
	Transfer	**0.05**	**0.07**	**0.49**	**0.50**	**0.32**	**0.35**	**0.19**	**0.21**	**0.04**	**0.05**

Notes: Mean and SD, the mean and standard deviation of mean squared errors (MSEs) of estimates; *n*, the number of correct scores in group G; *Ratio*, the percentage of top scores whose MSEs were evaluated

### Simulated MS/MS data

We designed a simulation experiment for identification of variant peptides, i.e. peptides containing single amino acid variations. The simulated MS/MS spectra used here were part of the data used in [19].

A total of 1,038,743 random tryptic peptide sequences were first generated. These peptides served as the non-variant peptides in the database to be searched. Then for each of these peptides, a variant peptide was generated by mutating one randomly selected amino acid of the peptide. Amino acids Isoleucine and Leucine were not allowed to be mutated between each other, and the peptide C-terminals were not allowed for mutation. The combination of these non-variant and variant peptides constituted the target database that was searched.

The simulated MS/MS spectra were composed of three parts: 20,000 variant spectra, 20,000 non-variant spectra and 80,000 noise spectra. The variant and non-variant spectra were theoretically generated from variant and non-variant peptides, respectively, which were randomly selected from the target database. The noise spectra were generated from additional sequences that were out of the target database.

In spectrum simulation, the mass-to-charge ratio (m/z) values of singly charged fragment ions of b and y types were predicted. The intensities of the fragment ions are randomly sampled from the uniform distribution. A number of noise peaks were generated and combined with fragment ions to form the MS/MS spectrum of the peptide. More details about the method to generate the simulated spectra can be found in Ref [14].

In each experiment, a dataset was constructed by including *n*(=1, 5, 10, 20, 50, 100) randomly selected variant spectra and 15000 randomly selected non-variant and noise spectra. The experiment was repeated 1000 times.

Mascot(v2.5.1) [27] was used as the search engine. Trypsin was specified as the proteolytic enzyme and no missed cleavage was allowed. The precursor and fragment mass matching tolerances were both 0.01 Da. No fixed or variable modifications were set for search. The database was searched in the target-decoy strategy by combining the target sequences with their reversed versions.

Figure 3 gives examples of the linear fitting results of *γ*_*G*_(*x*) and *λ*_*G*_(*x*). As shown, $\hat {\gamma }_{G}(x)$ is closely around the constant 0.5, and $\hat {\lambda }_{G}(x)$ is closely around the constant 0.1. Thus, it was assumed that *f*_*G*0_=*f*_0_ and *f*_*G*1_=*f*_1_ held.

**Fig. 4:**
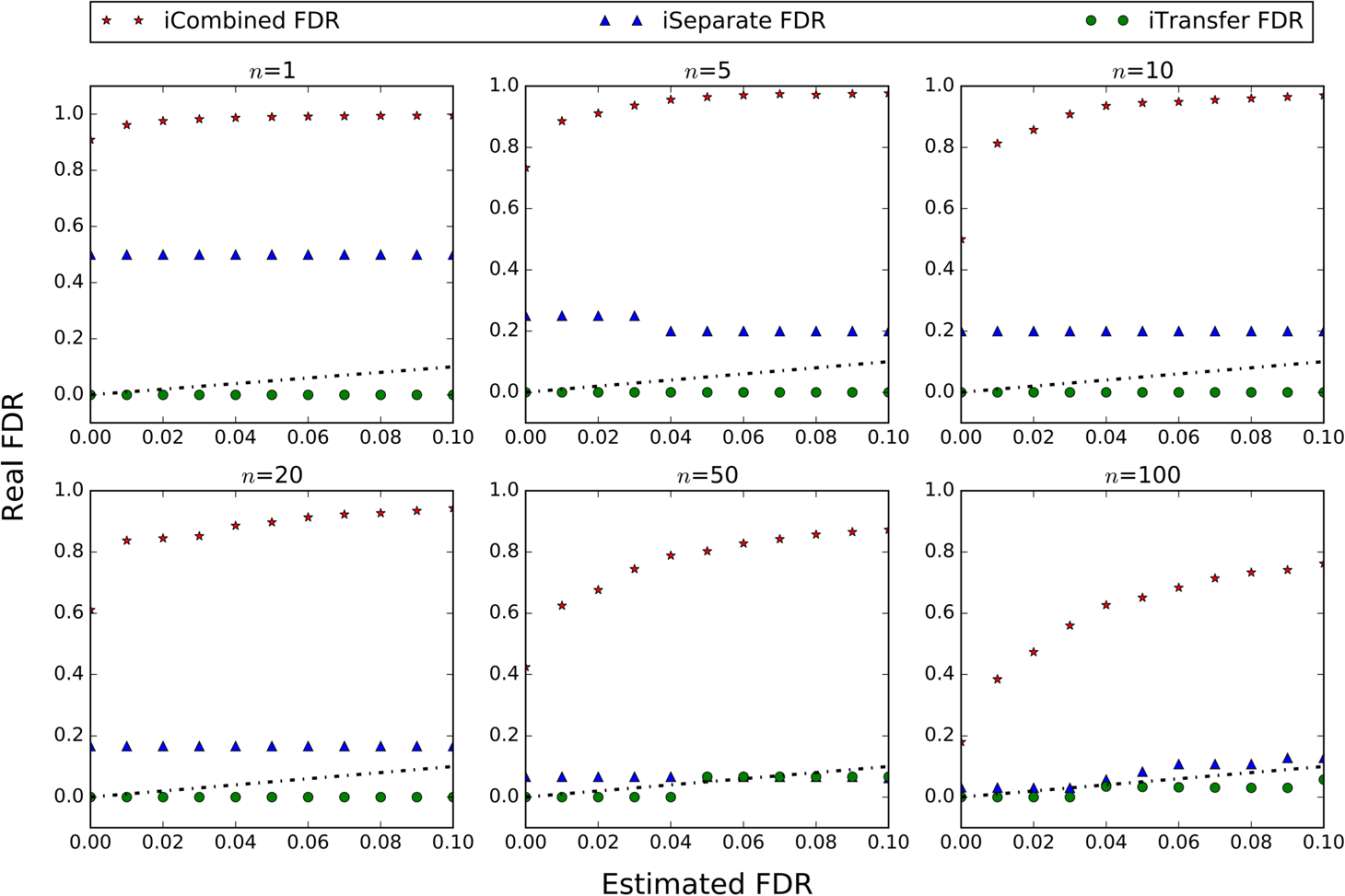
Comparison between the estimated and real variant FDRs at different FDR control level (1–10%) in the simulation study. The black dotted line represents *y*=*x*. Being close to or under the dotted line indicates that the FDR was successfully estimated or controlled

Figure 4 plots the estimated iCombined FDR, iSeparateFDR and iTransfer FDR against the real FDR at different FDR control levels (1–10%) for different group size *n* of variant spectra. As shown, iTransfer FDR was closest to the real FDR among the three estimates. Both iCombined FDR and iSeparate FDR remarkably deviated from the real FDR when the number of variant spectra was small. iSeparate FDR gradually approached to iTransfer FDR with increasing *n*, but iCombined FDR didn’t.

**Fig. 5:**
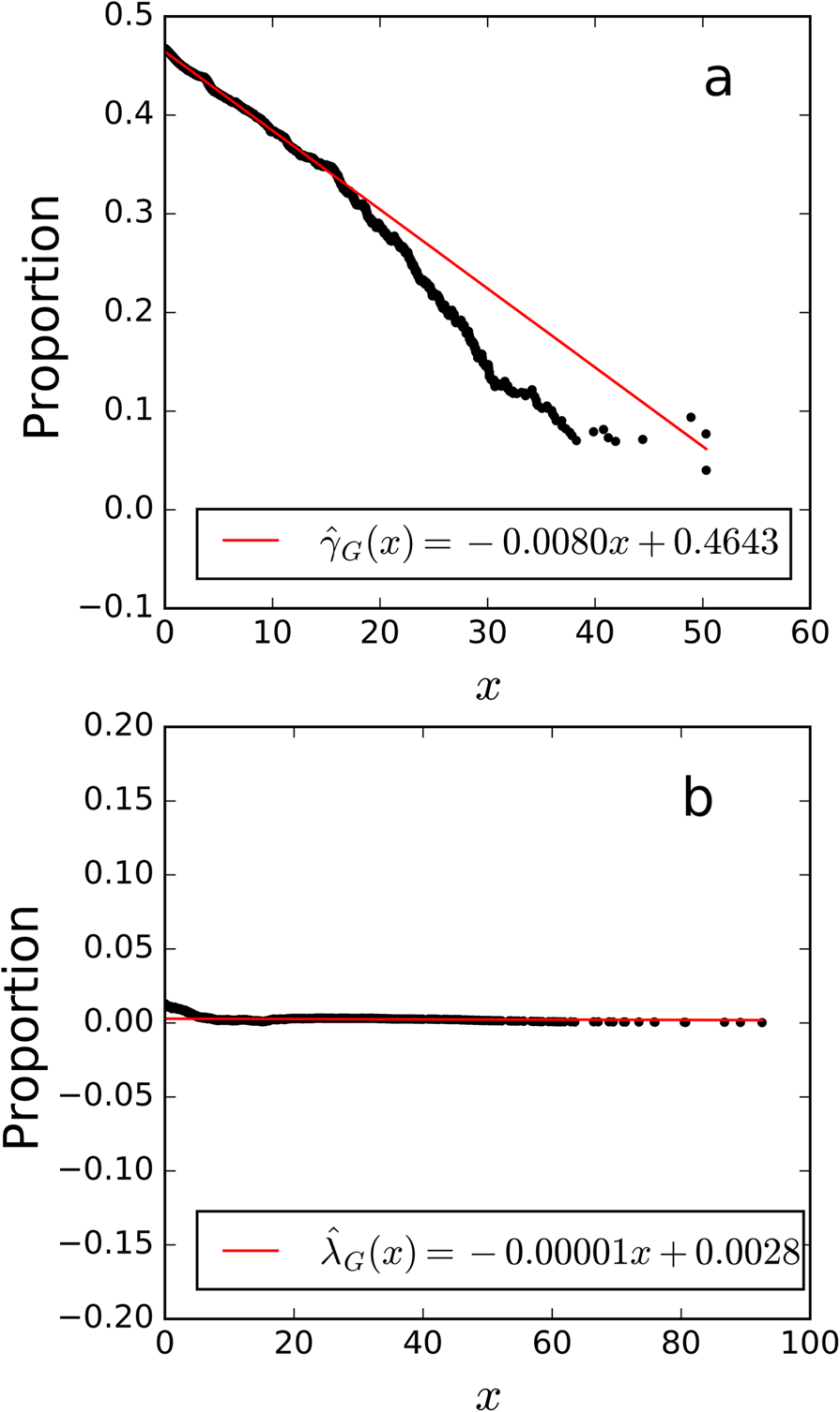
The linear fitting results of **a** *γ*_*G*_(*x*) and **b** *λ*_*G*_(*x*) on the human proteome draft dataset. The x-axis represents the score threshold, and the y-axis represents **a** the proportion decoy methylated PSMs among all decoy PSMs, or **b** the estimated proportion of correct methylated PSMs among all methylated PSMs above the score threshold *x*

Table 2 compares the three FDR estimation methods, in terms of the mean and SD of the estimation errors as well as the average numbers of all and false variant PSMs obtained at 1% FDR control level. As shown, iCombined FDR dramatically deviated from the real FDR. iSeparate FDR was much better, but still deviated from the real FDR when the number of variant spectra was small (*n*=1,5,10,20). The results of iCombined and iSeparate FDR gradually approached to those of iTransfer FDR with increasing *n*. iTransfer FDR was the best among these three methods for all the numbers of variant spectra.

**Table 2:** Results achieved with the three methods for estimating variant FDRs on simulated data, with the FDR control level at 1%

*n*	iCombined FDR			iSeparate FDR			iTransfer FDR		
	*Ave.#I.D.s*	*Est.error(%)*		*Ave.#I.D.s*	*Est.error(%)*		*Ave.#I.D.s*	*Est.error(%)*	
	*(false/all)*	*Mean*	*SD*	*(false/all)*	*Mean*	*SD*	*(false/all)*	*Mean*	*SD*
1	10.22/10.70	-94.36	5.13	0.21/0.55	-16.95	34.64	0.00/0.25	**0.00**	**0.02**
5	10.76/13.09	-80.95	8.84	0.30/2.05	-11.09	20.57	0.00/1.24	**0.01**	**0.07**
10	10.09/14.87	-66.43	9.50	0.60/4.17	-12.92	11.63	0.00/2.50	**0.04**	**0.14**
20	10.90/20.57	-51.64	9.08	0.72/ 8.07	-8.86	6.31	0.00/5.30	**0.08**	**0.19**
50	10.53/34.44	-29.25	6.87	0.73/19.07	-3.60	2.57	0.00/13.28	**0.20**	**0.27**
100	10.67/58.64	-17.09	4.54	0.73/38.27	-1.43	1.25	0.00/27.40	**0.42**	**0.35**

Note: *n*, the number of variant mass spectra; Ave.#I.D.s, average number of false/all identifications of variant peptides from the target database at 1% estimated FDR; Est.error, the difference between the estimated FDR and the real FDR; Mean and SD, mean and standard deviation of the Est.error as percentage

As the size of the group increases, the advantage of transfer PEP over other methods decreases. When the group size is large enough, the advantage vanishes. However, it is hard to say there is a fixed threshold at which the advantage disappears. It depends on the problem addressed, the dataset analyzed and other experimental conditions. According to our results, our method seems to be most effective when the group size is <50, and become comparable with other methods when the group size is >100.

### Real MS/MS data

In this section, we compared the three PEP estimation methods on a real MS/MS dataset. The objective judgement of the identification correctness is absent, so we used the transfer FDR [21] as the comparative reference. Two datasets were used for identification of methylated peptides and variant peptides, respectively.

#### Methylated peptide identification

The MS/MS spectra in this dataset were from the draft map of human proteome described in Kim et al. [28], and were downloaded from the PRIDE data repository (https://www.ebi.ac.uk/pride/, dataset identifier PXD000561). Briefly, this draft map was from protein samples of 30 human tissues which were analyzed on high-resolution Fourier-transform mass spectrometers using HCD fragmentation. In this paper, only the spectra of brain tissue were analyzed which included 24 RAW files.

Mascot(v2.5.1) [27] was used to identify the spectra. The protein sequence database searched was UniProt human protein database (v201506). All cysteines were assumed to be carbamidomethylated, and methionines were allowed to be oxidized. N-termini of peptides starting with glutamine residues were allowed to be pyroglutamined. N-termini of proteins were allowed to be acetylated. Both lysines and arginines were allowed to be methylated. Precursor and fragment mass matching tolerances were set as 10 ppm and 0.05 Da, respectively. Trypsin was specified as the proteolytic enzyme and up to two missed cleavages were permitted.

The linear fitting results of *γ*_*G*_(*x*) and *λ*_*G*_(*x*) are shown in Fig. 5. We can see that $\hat {\gamma }_{G}(x)$ varies in the interval [0,0.5], and $\hat {\lambda }_{G}(x)$ is almost constant at 0.0028. Thus, we assumed *f*_*G*0_≠*f*_0_ and *f*_*G*1_=*f*_1_.

**Fig. 6:**
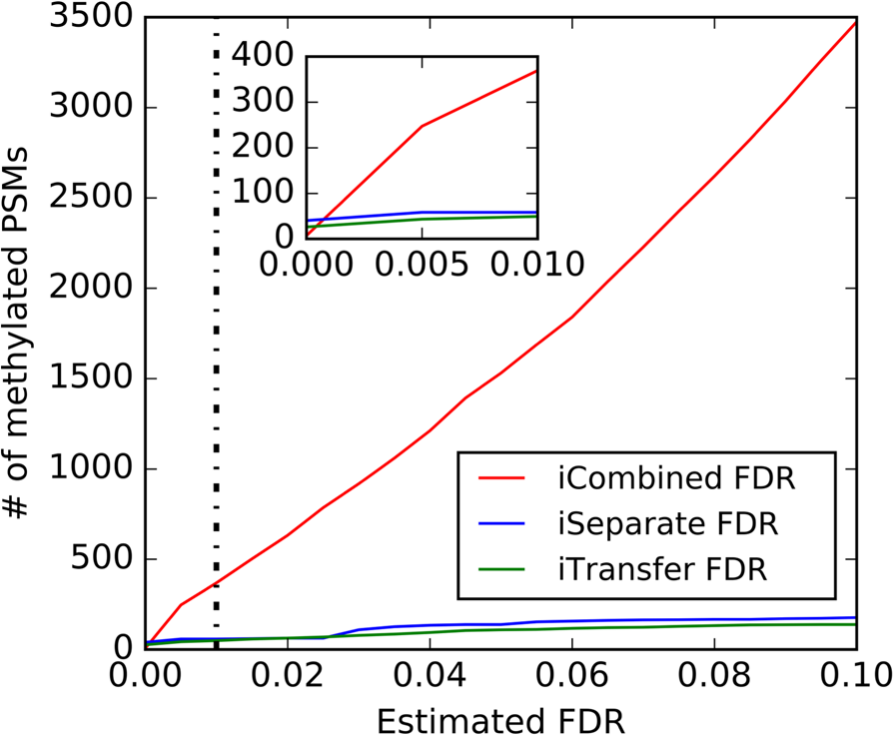
The numbers of methylated PSMs obtained with the three FDR methods at 0–10% FDR control level

**Fig. 7:**
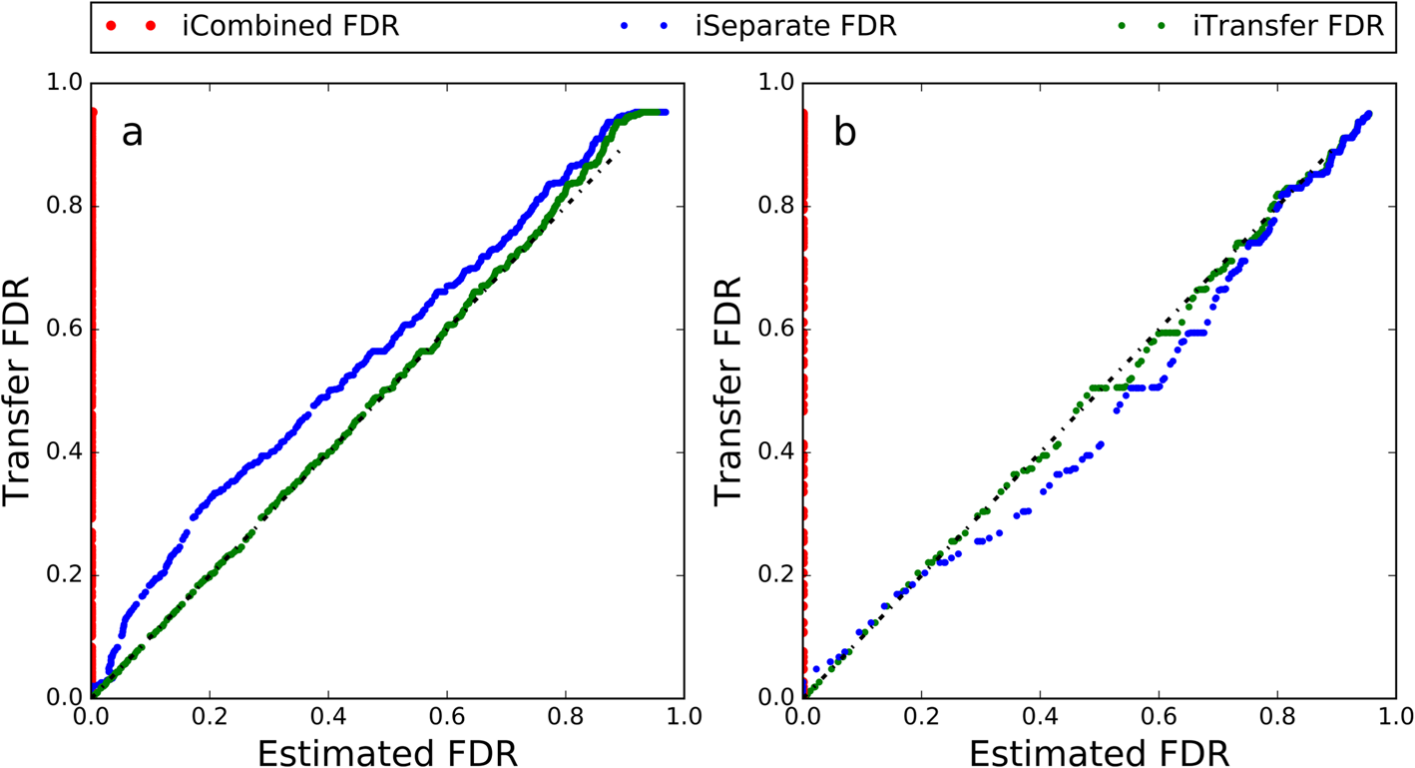
Comparison of the three FDR estimation methods with the transfer FDR in the **a** methylated peptides and **b** variant peptides identification studies

Figure 6 shows the numbers of identified methylated PSMs after filteration by the three FDR methods (iCombined FDR, iSeparate FDR and iTransfer FDR) at different FDR conrol levels (1–10%). Figure 7a shows the consistency of the three methods with transfer FDR. It is clear that iTransfer FDR was the most conservative and consistent with transfer FDR, iSeparative FDR was comparable but a little liberal, and iCombined FDR seriously underestimated the FDR.

**Fig. 8:**
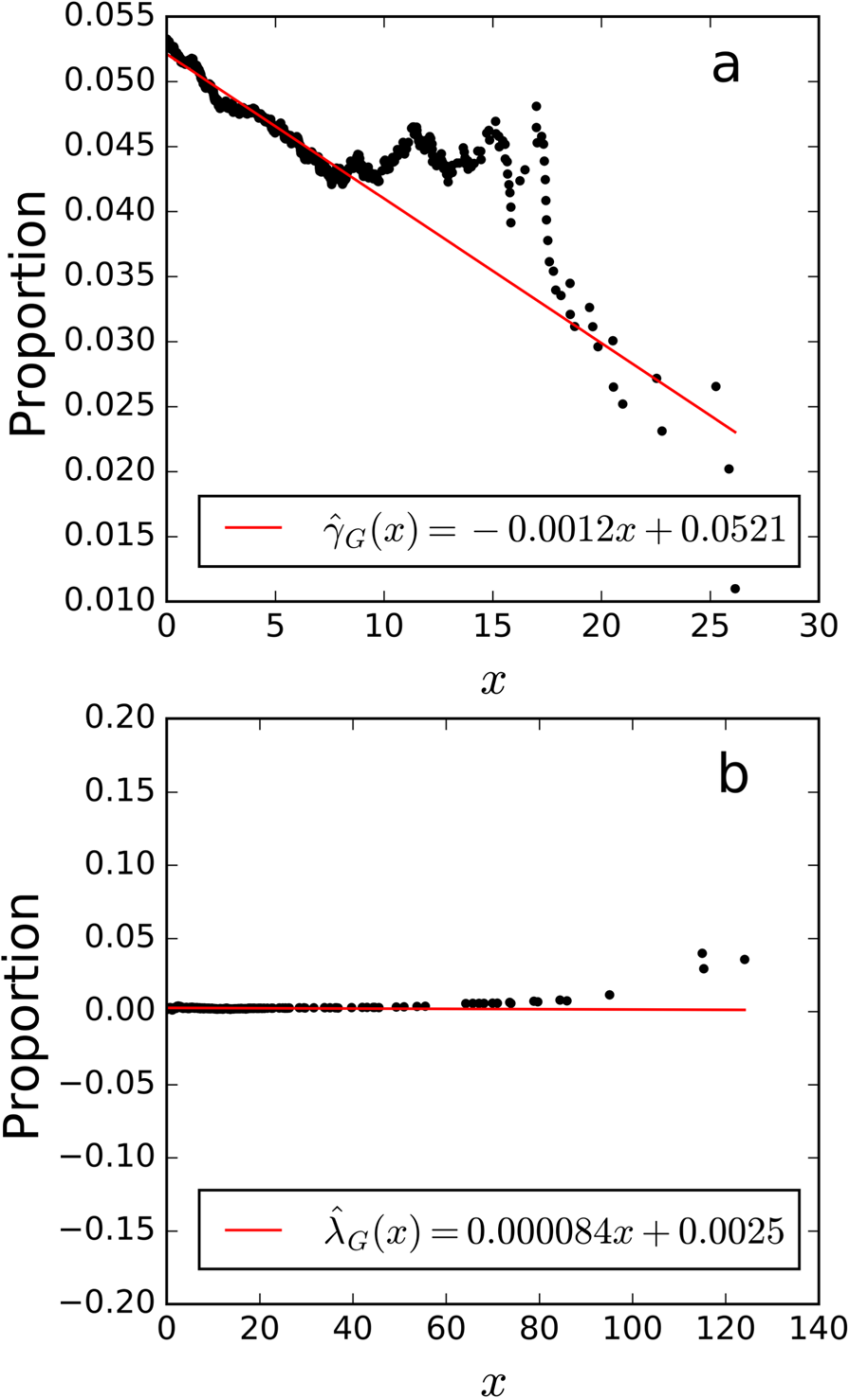
The linear fitting results of **a** *γ*_*G*_(*x*) and **b** *λ*_*G*_(*x*) on the colorectal cell line dataset. The x-axis represents the score threshold, and the y-axis represents **a** the proportion decoy variant PSMs among all decoy PSMs, or **b** the estimated proportion of correct variant PSMs among all variant PSMs above the score threshold *x*

#### Variant peptide identification

The data used for identification of variant peptides, i.e. peptides containing single amino acid variations, was part of a colorectal cell line dataset, which has been described in detail in Li et al. [24]. Proteins were digested by trypsin and analyzed on an LTQ-Orbitrap mass spectrometer. Only the spectra of SW480 sample were analyzed in this paper.

Mascot(v2.5.1) [27] was used to identify the spectra. The protein sequence database searched was MS-CanProVar(v1.0) [24], which can be downloaded from http://canprovar.zhang-lab.org/. All cysteines were assumed to be carbamidomethylated, and methionines were allowed to be oxidized. N-termini of peptides starting with glutamine residues were allowed to be pyroglutamined. Precursor and fragment mass matching tolerances were set as 10 ppm and 0.5 Da, respectively. Trypsin was specified as the proteolytic enzyme and two missed cleavages were permitted.

The linear fitting results of *γ*_*G*_(*x*) and *λ*_*G*_(*x*) are shown in Fig. 8. Accordingly, we assumed *f*_*G*0_≠*f*_0_ and *f*_*G*1_=*f*_1_ for this dataset. With the FDR control level set at 1%, 42, 36 and 32 variant PSMs were obtained by iCombined, iSeparate, and iTransfer FDRs, respectively. Figure 7b shows that, similar to the result of methylated peptide identification, iTransfer FDR was the most consistent with transfer FDR.

## Conclusions

In this paper, we have presented transfer PEP, the first solution to the problem of PEP estimation for small groups of peptide identifications in proteomics. By using the empirical relationship between the combined null distribution and the group null distribution of identification scores, transfer PEP makes possible accurate PEP estimation for data of very limited sample size. The small groups are not uncommon in proteomics. For example, when one focuses on identifying amino acid mutations [19] or open searching of PTMs [22, 29], the concerned group is often very small, typically <50. Given the group null distribution, transfer PEP uses an iterative semi-parametric method to estimate the group alternative distribution and the null proportion. Because kernel density estimation is used, transfer PEP does not require the distribution forms to be known and thus is applicable to different scoring functions. The performance of transfer PEP was validated on both the simulated data and the real mass spectra datasets. Compared with the combined and separate PEPs, transfer PEP showed much more accuracy in estimating the PEP of small groups without loss of power. Estimation of PEP enables evaluation of the confidence of individual peptide identifications, which is desirable in many circumstances, e.g. protein inference [30]. Finally, it is worthwhile to note that transfer PEP is in principle adaptable to the small-group PEP estimation problems in other fields, as long as *γ*_*G*_(*x*) can be estimated, which is not limited to the linear form.

## Data Availability

The transfer PEP algorithm was implemented in Matlab. The source codes and the test data are available at http://fugroup.amss.ac.cn/software/TransferPEP/TransferPEP.html. The peptide mass spectra we used are publicly available.

## Abbreviations

PSM: peptide-spectrum match
FDR: false discovery rate
PEP: posterior error probability
MS/MS: mass spectrometry
pdf: probability density function
EM: Expecting Maximization
PTM: post-translational modification
cdf: cumulative distribution function
SD: standard deviation
MSE: mean squared error
m/z: mass-to-charge ratio
